# Antibacterial Activity of a Fused Endolysin ENDO‐1252/KL9P Against Multiple Serovars of *Salmonella enterica*

**DOI:** 10.1111/1751-7915.70237

**Published:** 2025-10-08

**Authors:** Chuan‐Wei Tung, Kanchan Thapa, Anna Phan, Aditi Mohapatra, Muhammad Hashmi, Kayla Bleich, Debabrata Biswas

**Affiliations:** ^1^ Department of Animal and Avian Sciences University of Maryland College Park Maryland USA; ^2^ Biological Sciences Program‐Molecular and Cellular Biology University of Maryland College Park Maryland USA; ^3^ Department of Neuroscience University of Maryland College Park Maryland USA

**Keywords:** antibiotic resistance, bacteriophage, endolysin, food safety, fused protein, *Salmonella enterica*, *Salmonella enteritidis*

## Abstract

*Salmonella enterica* (SE) is one of the most prevalent enteric pathogens globally and infects humans through contaminated food and water sources. The rising trend of antibiotic‐resistant SE strains poses a critical threat to public health. Bacteriophage‐encoded endolysins evolve a promising alternative as antimicrobial agents for combating SE infections. These enzymes target the peptidoglycan layer of bacterial cells, causing cell lysis and death. However, the use of endolysins against Gram‐negative bacteria is challenging due to the composition of the outer membrane, which acts as a barrier preventing the endolysins from reaching the peptidoglycan layer. KL9P is a short amphipathic peptide containing both hydrophobic and hydrophilic regions, enabling it to interact with membranes and aqueous environments. In this study, an endolysin ENDO‐1252, a *Salmonella* bacteriophage‐encoded enzyme, was fused with a short peptide KL9P and produced an advanced endolysin, ENDO‐1252/KL9P, which enhanced its ability to lyse multiple serovars of SE. ENDO‐1252/KL9P exhibited potent lytic activity against SE strains with optimal bactericidal effects observed at 20 μM and incubation at 37°C in 20 mM HEPES buffer (pH 7.4). The lytic activity of this endolysin was also evaluated under various conditions, including pH ranges and temperatures, revealing that ENDO‐1252/KL9P retained significant lytic activity across a range of temperatures (25°C–40°C) and pH values (6.0–9.0). The fusion protein demonstrated the highest lytic efficiency against SE serovars, specifically *S.* Enteritidis, *S.* Heidelberg, and *S.* Pullorum. Immunofluorescence analysis confirmed the binding of ENDO‐1252/KL9P to the bacterial cell wall, indicating the co‐localization with the peptidoglycan layer. These results suggest that ENDO‐1252/KL9P is a promising antibacterial agent inhibiting predominant serovars of SE, showing enhanced lytic activity without outer membrane permeabilizers.

## Graphical abstract

Schematic overview of the construction of fusion endolysin ENDO‐1252/KL9P. (A) The gene for endolysin from the Salmonella phage‐1252 gene (GenBank ID: PP695294.1) was amplified by PCR from the genomic DNA, along with primers containing the endolysin‐linker residues. (B) The amplified endolysin‐linker gene was further re‐amplified using a forward primer containing the linker and KL‐L9P residues (KLLKLLKKPLKLLK). Subsequently, a second PCR was performed and created an overlap gene. (C) The purified fusion gene products were ligated into the plasmid pET‐28c, which carries a C‐terminal 6× histidine (His)‐tag sequence. (D) The recombinant plasmids containing the fusion gene were transformed into *E. coli* BL21(DE3). (E) Schematic representation of the fusion endolysin ENDO‐1252/KL9P, including the linker and 6×His tag.

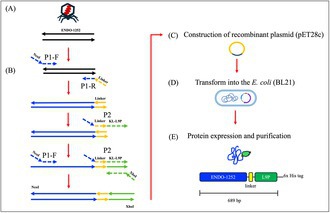

## 1 Introduction

*Salmonella enterica* (SE), especially nontyphoidal SE, is recognised as the most common foodborne zoonotic pathogen worldwide (World Health Organization 2016; Chattaway et al. 2021). On the other hand, decades of excessive and inappropriate use of antimicrobials both in humans and animals have caused a widespread increase in antimicrobial resistance (AMR) bacterial pathogens, which poses an additional threat to treat bacterial diseases (Aditya et al. 2023, 2024; Alvarado‐Martinez, Julianingsih, et al. 2023; Naghavi et al. 2024). Researchers aimed to develop alternative strategies to control the colonisation of SE, such as citrus oil (Julianingsih et al. 2023), plant‐derived phenolics (Alvarado‐Martinez, Tabashsum, et al. 2023; Thapa et al. 2024), bacteriophages (Tung et al. 2023), and phage‐encoded endolysins (Schmelcher et al. 2012; Tung et al. 2024). Among all the alternatives against SE, we focused on endolysins as a potential option. However, their effectiveness may have been limited, likely due to the complex cell wall structure of Gram‐negative bacteria and the strain diversity within SE. (Tung et al. 2024). Therefore, we aimed to develop an effective endolysin against multiple serovars of SE in this study.

Endolysins are muralytic enzymes that degrade the peptidoglycan layer of the bacterial cell wall (Gondil et al. 2020). These enzymes are integral to the lytic cycle of bacteriophage infection. Endolysins show significant potential as a solution against various bacterial pathogens with antimicrobial resistance (Gondil et al. 2020; Rahman et al. 2021). Because some endolysins display species‐ or strain‐specific activity, they are often considered microbiome‐friendly, with the potential to selectively target pathogenic bacteria in the ecosystem or the human and animal gut (Schmelcher et al. 2012; Pottie et al. 2024). However, it is important to note that not all endolysins are highly specific, while some exhibit broader range of activity, which may impact beneficial microbiota unless engineered for enhanced specificity. Additionally, endolysins rapidly lyse bacterial cells, have a minimal risk of inducing resistance, are effective in synergistic function with other antibacterial agents, and can effectively fight against biofilms and mucosal surfaces (Schmelcher et al. 2012). Due to these benefits, many purified or recombinant endolysins have been used to address multidrug‐resistant bacteria, specifically Gram‐positive, making them a promising alternative treatment (Rahman et al. 2021). Since the discovery of the first endolysin from the *Listeria monocytogenes* phage in 1995, endolysins have generated interest as a new class of natural food preservatives in the market (Loessner et al. 1995).

Compared to Gram‐positive bacteria, the applications of endolysins against Gram‐negative bacteria remains limited, even though some endolysins exhibit activity are against them. This limitation is due to the structural complexity of the Gram‐negative cell wall, which consists of an outer membrane with a lipopolysaccharide (LPS) layer that prevents endolysins from accessing and lysing the peptidoglycan layer (Loessner 2005). So far, only a few endolysins showed some effectiveness against Gram‐negative bacteria, such as SE (Lim et al. 2012; Legotsky et al. 2014; Li et al. 2016; Jiang et al. 2021), *Vibrio parahaemolyticus* (Wang et al. 2016), *Shewanella putrefaciens* (Han et al. 2014), and *Pseudomonas aeruginosa* (Guo et al. 2017). To enhance the effectiveness of the endolysins against Gram‐negative bacteria, it is necessary to utilise outer membrane permeabilizers (OMPs), such as organic acids (Rahman et al. 2021), ethylenediaminetetraacetic acid (EDTA) (Liu et al. 2019), trichloromethane (Vaara 1992), Triton X‐100 (Vaara 1992) or the edible ε‐poly‐L‐lysine (EPL) (Han et al. 2019), to either pretreat or co‐treat the bacteria when applying exogenous endolysins.

Sensitizer peptides enhance the permeability of the outer membrane of Gram‐negative bacteria, potentially allowing antibiotics, typically effective against Gram‐positive bacteria, to be used against Gram‐negative bacteria (Son et al. 2023). Sensitizer peptides work by remodelling the anionic LPS molecules in the outer membrane of Gram‐negative bacteria, thereby compromising the impermeability of the outer membrane to antimicrobial agents. Unlike conventional cell‐penetrating peptides, sensitizer peptides do not disrupt or penetrate the membrane. Instead, they can alter the membrane structural integrity and function of the outer membrane, making it more permeable to antimicrobial agents, such as antibiotics or endolysins (Hyun et al. 2020).

Recently, researchers found that KL‐L9P, a pro‐hinged α‐helical amphipathic sensitizer peptide, remodels the outer membrane without damaging the outer and inner membranes (Hyun et al. 2020). It enables many hydrophobic antibiotics to become effective against Gram‐negative bacteria, including AMR bacterial pathogens (Hyun et al. 2020). Based on these properties and previous studies (Hyun et al. 2020; Son et al. 2023; Tung et al. 2024), we hypothesised that ENDO‐1252 fused with KL‐L9P could efficiently pass through the impermeable outer membrane of SE. Following the approach from a previous study (Son et al. 2023), we aim to construct a fused endolysin protein, ENDO‐1252, with KL‐L9P and enhance its antimicrobial activity without OMP permeable agent. We subsequently evaluated its lytic activity against multiple serovars of SE.

## 2 Materials and Methods

### 2.1 Bacteriophage, Bacterial Strains and Culture Conditions

**FIGURE 1:**
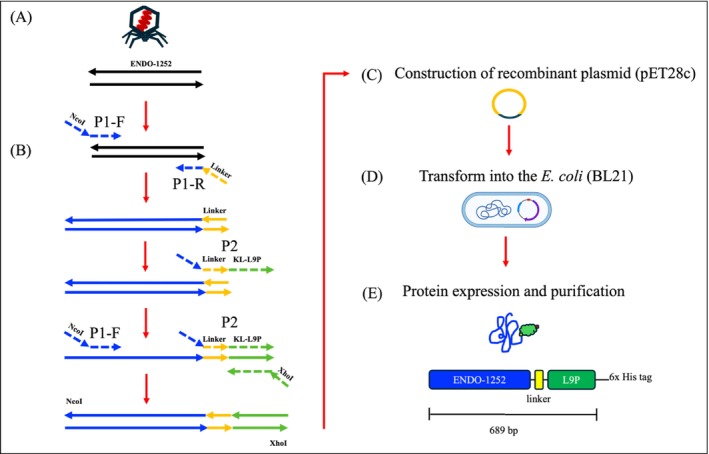
Schematic overview of the construction of fusion endolysin ENDO‐1252/KL9P. (A) The gene for endolysin (87,728–88,345) from the Salmonella phage‐1252 gene (GenBank ID: PP695294.1) was amplified by PCR from the genomic DNA using primers with NcoI overhangs at the N‐terminal end, along with primers containing the endolysin‐linker residues. (B) The amplified endolysin‐linker gene was further re‐amplified using a forward primer containing the linker and KL‐L9P residues (KLLKLLKKPLKLLK). Subsequently, a second PCR was performed with the forward primer ENDO‐1252 NcoI_F and reverse primer KL‐L9P‐XhoI_R, both containing NcoI and XhoI overhangs, respectively, to create an overlap gene. (C) The purified fusion gene products were digested double by restriction enzymes NcoI and XhoI, then ligated into the plasmid pET‐28c, which carries a C‐terminal 6× histidine (His)‐tag sequence. (D) The recombinant plasmids containing the fusion gene were transformed into *E. coli* BL21(DE3). (E) Schematic representation of the fusion endolysin ENDO‐1252/KL9P, including the linker and 6×His tag.

The bacterial strains used in this study and their sources are listed in Table 1. Genomic DNA of *Salmonella* bacteriophage‐1252 (phage‐1252, GenBank ID: PP695294.1), isolated, characterised, and reported by Tung et al. (2023), was extracted. SE serovar Enteritidis ATCC 13076 (*S*. Enteritidis) was used as the host strain for propagation of phage‐1252 and for evaluating the lytic activity of this endolysin (Figure 1A). All serovar of SE (Enteritidis, Typhimurium, Heidelberg, Kentucky, Gallinarum, and Pullorum) were grown on Luria–Bertani (LB) agar or in broth (Becton, Dickinson and Co., Sparks, MD, USA) at 37°C overnight. *E. coli* BL21, containing plasmid pET28c, was used as a vector and grown in LB broth or on LB agar with kanamycin (50 μg mL^−1^). For *S*. Enteritidis 13076 culture conditions, cell culture preparations were made from a plate and sub‐cultured in LB broth, incubated at 37°C overnight, then diluted (1:100) and incubated at 37°C for 3 h. Subsequently, the OD_600_ was measured to 0.1 using a spectrometer in 200 μL of LB broth.

**TABLE 1:** *Salmonella* strains were used in this study.

Strain	Sources	References
*Salmonella enterica* serovar Typhimurium	ATCC STLT2	Tung et al. (2023)
*Salmonella enterica* serovar Enteritidis	ATCC 13076	Tung et al. (2023)
*Salmonella enterica* serovar Kentucky	Farm samples	Tung et al. (2023)
*Salmonella enterica* serovar Heidelberg	Farm samples	Tung et al. (2023)
*Salmonella enterica* serovar Pollurum	ATCC 19945	Tung et al. (2023), Julianingsih et al. (2024)
*Salmonella enterica* serovar Gallinarum	ATCC 9184	Tung et al. (2023), Julianingsih et al. (2024)
*Salmonella enterica* serovar Newport	Farm samples	Tung et al. (2023)

### 2.2 Cloning to Construct the Fused Genome Structure

The fused genome structure was constructed by overlapping Linkers (GGGGS) and KL‐L9P (KLLKLLKKPLKLLK) residues using the polymerase chain reaction (PCR) following the method previously described by (Son et al. 2023) with minor modifications. In brief, the endolysin gene of phage‐1252 (GenBank ID: PP695294) (Tung et al. 2024) was amplified by PCR using primers with NcoI overhanging ends at the N‐terminal side (ENDO‐1252 NcoI_F) and primers with endolysin‐Linker residues (ENDO‐1252 Link_R). Endolysin with Linker gene products were then re‐amplified with forward primer ENDO‐1252 NcoI_F and forward primer with Linker and KL‐L9P residues (ENDO‐1252 Link‐KL‐L9P_F) in PCR for 12 cycles. Subsequently, forward primer ENDO‐1252 NcoI_F and reverse primer KL‐L9P‐XhoI_R with XhoI overhanging ends were added, and PCR was continued for 18 cycles to construct the overlap gene (Figure 1B). The primers used in this study are listed in Table 2.

**TABLE 2:** Primers used in this study.

Primer	Sequence (5′–3′)
ENDO‐1252 NcoI_F	CGCGCCCATGGGCGCGATGACCCTATCAACTCAGGAGGTT
ENDO‐1252 Link_R	GCTGCCGCCGCCGCCTTTGCACATCCCCAACTT AGCCACGTAG
ENDO‐1252 Link‐KL‐L9P_F	AAGTTGGGGATGTGCAAAGGCGGCGGCGGCAGCAAACTGCTGAAACTGCTGAA GAAACCGCTGAAACTGCTGAAA
KL‐L9P‐XhoI_R	GCGCTCGAGCGGTTTCAGCAGTTTCAGCGGTTT CTTCAGCAGTTTCAGCAGTTTGCTGCCGCCGCC GCCTTTGCACATCCCCAACTTAGCCACGTAG
Link‐KL‐L9P F	CGCGCCCATGGGCGCGATGGGCGGCGGCGGCAGCAAACTGCTG
Link‐KL‐L9PR	GCGCTCGAGCGGTTTCAGCAGTTTCAGCGGTTTCTTCAGC

The fused genome products were purified using a PCR purification kit (Invitrogen, Thermo Fisher Scientific IL, USA), then double digested by restriction enzyme NcoI and XhoI and ligated into the plasmid pET‐28c (Novagen, Madison WI, USA) with a C‐terminal 6× histidine (His)‐tag sequence (Figure 1C). Subsequently, the recombinant plasmids with fusing genome products were transformed into the *E. coli* BL21(DE3) and selected with kanamycin (50 μg mL^−1^) in LB agar plates (Figure 1D). Selected colonies were confirmed by PCR with primer ENDO‐1252 Link‐KL‐L9P_F and KL‐L9P‐XhoI_R.

### 2.3 Protein Overexpression and Purification

The methods of protein overexpression and purification were followed by the method previously described by Tung et al. (2024) (Tung et al. 2024). Briefly, the overexpressed of *E. coli* BL21(DE3) with plasmid were lysed and purified by HisPur Ni‐NTA Resin (Thermo Scientific, MA, USA). For purification, equilibrated buffer (20 mM Imidazole, 350 mM NaCl, 1% Tween‐80, and 20 mM Tris–HCl pH 7.4) and 50 mM Imidazole buffer (50 mM Imidazole, 350 mM NaCl, 0.002% Tween‐80, and 20 mM Tris–HCl pH 7.4) were used. Subsequently, the His‐tagged recombinant protein was eluted with 300 mM Imidazole buffer (300 mM Imidazole, 350 mM NaCl, 0.002% Tween‐80, and 20 mM Tris–HCl pH 7.4). The eluted protein was concentrated with an Amicon Ultracel 10,000 MCWO concentrator (Millipore, MD, USA). The elution protein was exchanged to the storage buffer (350 mM NaCl, 0.002% Tween‐80, and 20 mM Tris–HCl pH 7.4) with Dialysis sacks (Sigma‐Aldrich, MO, USA) for further testing (Figure 1E). The protein concentration was determined using the BCA Protein Assay Kit (Thermo Scientific, MA, USA).

### 2.4 SDS‐PAGE and SDS‐PAGE Western Blotting

The purified protein was analysed using sodium dodecyl sulphate‐polyacrylamide gel electrophoresis (SDS‐PAGE), followed by protein blotting (Garić et al. 2023). Briefly, the gel was equilibrated in Towbin buffer (25 mM Tris, 192 mM glycine, 20% (v/v) methanol, 0.025% SDS, pH 8.3) for 15 min and transferred onto a nitrocellulose membrane (0.45 μm Pore Size) (Novex, CA, USA) using the Trans‐Blot SD Semi‐Dry Transfer Cell (Bio‐Rad, CA, USA) at 25 V for 120 min. Then the membrane was washed thoroughly with deionised (DI) water, blocked with 2% BSA in PBS by incubating at 4°C overnight. After blocking, add a 1:3000 dilution of 6×‐His Tag Monoclonal Antibody (Invitrogen, Thermo Fisher Scientific, IL, USA) in 2% BSA by incubating for 3 h at room temperature. After washing with PBS, the membrane was re‐incubated with a 1:2000 dilution of Goat anti‐Mouse IgG2b Cross‐Adsorbed Secondary Antibody, HRP (Invitrogen, Thermo Fisher Scientific, IL, USA) in 0.1% BSA in PBS for 1 h at room temperature. Finally, the membrane was washed and incubated with HRP working solution (SuperSignal West Dura, Invitrogen, Thermo Fisher Scientific, IL, USA) for 5 min. Chemiluminescent detection was performed using the Azuro 300 Chemiluminescent Western Blot Imager (Azure Biosystems Inc., CA, USA).

### 2.5 Determine the Bactericidal Activity of ENDO‐1252/KL9P Under Different Buffer Systems and pH Levels

Approximately 10^6^ CFU mL^−1^of *S*. Enteritidis was prepared and resuspended with a target buffer, such as Tris–HCl (1 M Tris–HCl pH 8.0 ± 0.1, Mediatech Inc., VA, USA), HEPES (1 M HEPES Buffer, MP biomedicals, OH, USA), or autoclaved distilled water. Afterward, 2 μL of ENDO‐1252/KL9P (500 μg mL^−1^) was added to 50 μL of the resuspended bacterial culture and incubated at 37°C for various time points (1, 3, 6, 9, and 12 h). Survived *S*. Enteritidis cells were determined by serial dilution and plating on LB agar. As a negative control, an equivalent volume of elution buffer was added instead of ENDO‐1252/KL9P. For the effect of the pH on the lytic activity of ENDO‐1252/KL9P, the target buffer adjusted its pH levels using HCl or NaOH to achieve targeted pH levels of 6.0, 7.4, 8.0, and 9.0 before performing the lytic assay described above.

### 2.6 Dose‐Dependent Antibacterial Activity of ENDO‐1252/KL9P

A volume of 50 μL *S*. Enteritidis culture (containing approximately 10^6^ CFU mL^−1^) was prepared and 0, 2.5, 5, 10, 20, and 40 μM of ENDO‐1252/KL9P (500 μg mL^−1^) was added. The samples were incubated at 37°C for 6 h, followed by serial dilution and plating on LB agar. As a negative control, an equivalent volume of elution buffer was added instead of ENDO‐1252/KL9P.

### 2.7 Stability and Effect of Temperature on Bactericidal Activity of ENDO‐1252/KL9P

A volume of 50 μL *S*. Enteritidis culture (containing approximately 10^6^ CFU mL^−1^) in HEPES (pH 7.4) was prepared, and 20 μM of ENDO‐1252/KL9P (500 μg mL^−1^) was added. The samples were incubated at 4°C, 15°C, 25°C, 37°C, 40°C, and 50°C for 6 h, followed by serial dilution and plating on LB agar. As a negative control, an equivalent volume of elution buffer was added instead of ENDO‐1252/KL9P.

### 2.8 Comparison of Antibacterial Activity Between Fused Endolysin ENDO‐1252/KL9P and Native ENDO‐1252 Against Various SE Serovars

Various SE serovars (Table 1) were grown overnight in LB broth at 37°C. A volume of 50 μL SE serovar (each serovar separately) culture (containing approximately 10^6^ CFU mL^−1^) was prepared and treated by either native form 20 μM ENDO‐1252, 0.1 mM EDTA, native form 20 μM ENDO‐1252 with 0.1 mM EDTA combination, or 20 μM fusion endolysin ENDO‐1252/KL9P. The samples were incubated at 37°C for 6 h, followed by serial dilution and plating on LB agar. As a negative control group, an equivalent volume of protein elution buffer or autoclaved distilled water was added in place of the treatments.

### 2.9 Confocal Laser Fluorescence Microscopy Image

The protocol for staining *S*. Enteritidis and ENDO‐1252/KL‐L9P with the FDAA 7‐hydroxycoumarincarbonylamino‐D‐alanine (HADA, Tocris Bioscience, U.K.) and double immunofluorescence was followed with modifications as previously described by Peters et al. (2019). Briefly, 600 μL *S*. Enteritidis culture was treated with a final concentration of 250 μM HADA stock solution and incubated at 37°C for 30 min. Then, the cells were washed with HEPES (pH 7.4) and treated with either 20 μM ENDO‐1252 or 20 μM ENDO‐1252/KL‐L9P at 37°C for 1 h, and then sodium citrate buffer in various pH levels was added. The cells were centrifuged, and pellets were incubated with 4% paraformaldehyde for 10 min at 37°C. The cells were washed with PBS and blocked with 1 mL 2% BSA in PBS and incubated for 1 h at room temperature. Discarding the blocking buffer by centrifugation, the cells were suspended in PBS containing 0.1% BSA with 6×‐His Tag Monoclonal antibody (1:500) and incubated for 3 h at room temperature. After washing with PBS, the cells were resuspended in PBS containing 0.1% BSA with Goat anti‐Mouse IgG2b Cross‐Adsorbed Secondary Antibody (1:1000), Alexa Fluor 555 (Invitrogen, Thermo Fisher Scientific IL, USA) was added in 0.1% BSA in PBS and incubated for 1 h at room temperature protected from light. Then, the antibody solution was removed and washed with PBS, and resuspended with SlowFade Gold antifade reagent (Invitrogen, Thermo Fisher Scientific IL, USA). The cells were fixed and observed using a microscopic agarose slide (1% w/v in PBS) and analysed the labelled cells with STELLARIS 8 FALCON FLIM Microscope.

### 2.10 Statistical Analysis

One‐way and two‐way analyses of variance (ANOVA) were conducted, followed by Tukey's multiple‐comparison test for post hoc comparisons with the control group. Statistical analyses were performed using Python's SciPy library, with a significance threshold set at 0.05, 0.005, and 0.001. Each experiment was independently replicated three times (*n* = 3), and results are presented as mean ± standard deviation.

## 3 Results

### 3.1 Characteristics of Fused Endolysin ENDO‐1252/KL9P

**FIGURE 2:**
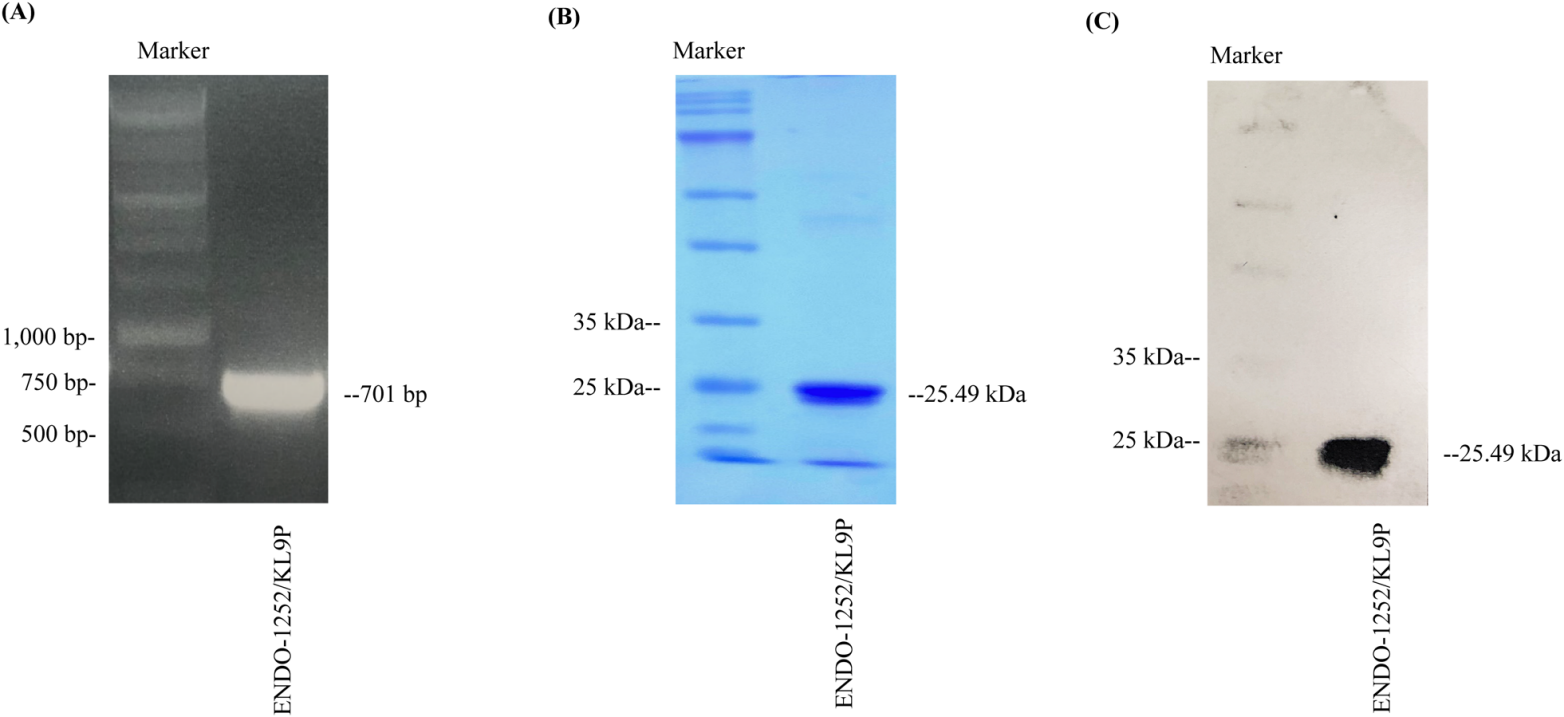
Confirmation of ENDO‐1252/KL9P gene construction, expression, and purification. (A) PCR confirmation of the ENDO‐1252/KL9P DNA fragment (701 bp) extracted from recombinant *E. coli* BL21 containing the ENDO‐1252/KL9P gene in the pET28c plasmid; Lane 1: DNA ladder (Marker). (B) SDS‐PAGE analysis showing the successful overexpression and purification of the ENDO‐1252/KL9P fusion protein; Lane 1: Protein molecular weight marker; Lane 2: Eluted (purified) ENDO‐1252/KL9P fusion protein. (C) SDS‐PAGE protein blotting confirmed fusion protein ENDO‐1252/KL9P (25.49 kDa). Lane 1: Protein molecular weight marker; Lane 2: Eluted (purified) ENDO‐1252/KL9P fusion protein.

The PCR product of the fused endolysin ENDO‐1252/KL9P was generated using overlap extension, ligated into the pET‐28c plasmid (containing a C‐terminal 6× histidine tag), and subsequently transformed into *E. coli* BL21(DE3) (Figure 1). PCR and SDS‐PAGE analysis showed a single band around 700 bp on the PCR gel (Figure 2A) and 25 kDa on the SDS‐PAGE (Figure 2B), consistent with the predicted molecular weights of ENDO‐1252/KL9P (701 bp for DNA and 25.49 kDa for protein). The successfully purified fusion endolysin ENDO‐1252/KL9P was reached at a concentration of 3.6 mg mL^−1^ from a litre culture.

### 3.2 Bactericidal Activity of ENDO‐1252/KL9P at Various Conditions

**FIGURE 3:**
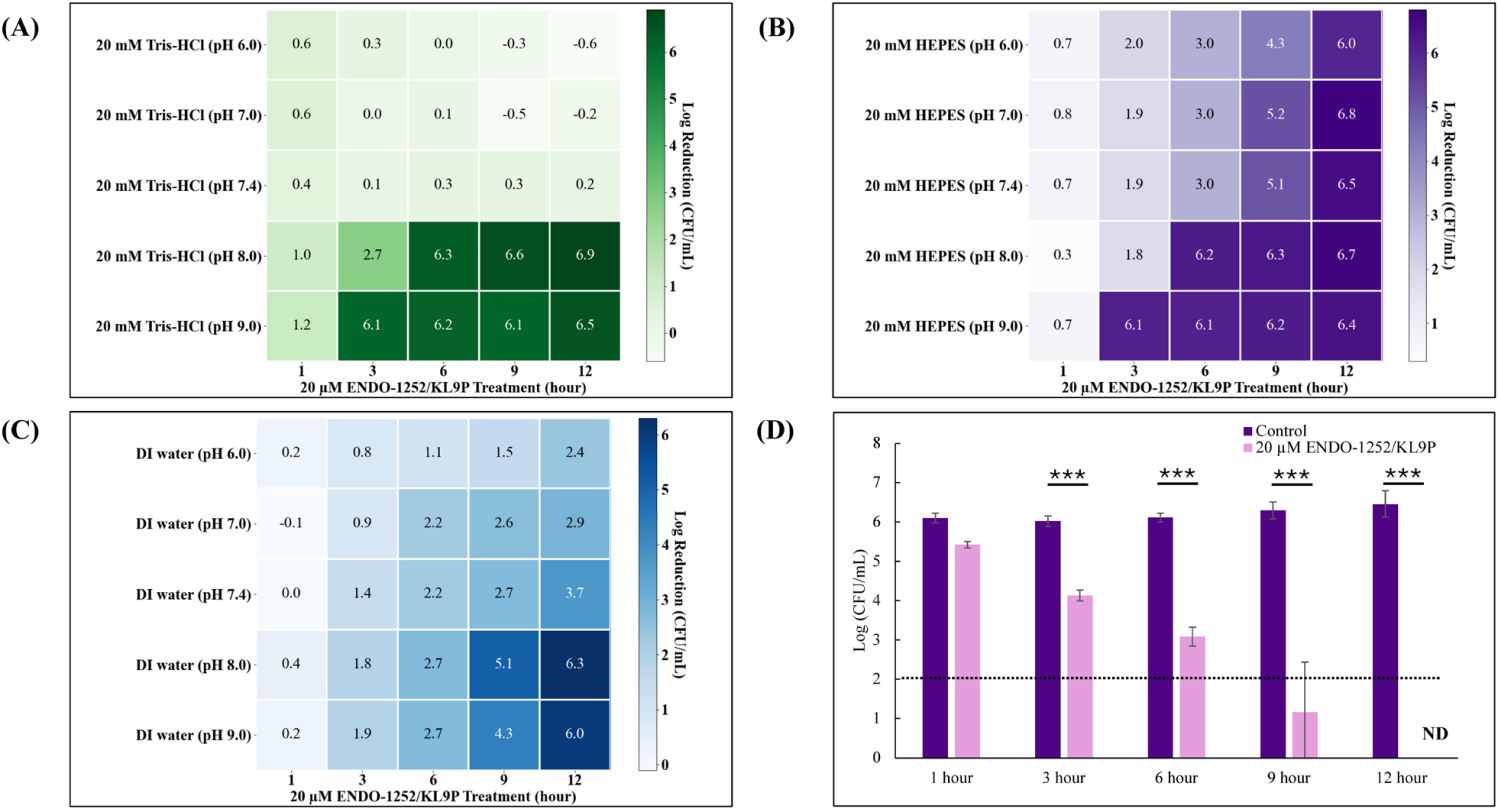
Checkerboard assay of antibacterial activity of the fusion ENDO‐1252/KL9P across different buffer conditions and pH levels. (A) *S*. Enteritidis was treated with the fusion ENDO‐1252/KL9P and incubated at 37°C for various time points in 20 mM Tris–HCl buffer, with pH levels ranging from 6.0 to 9.0. (B) *S*. Enteritidis was treated with the fusion ENDO‐1252/KL9P and incubated at 37°C for various time points in 20 mM HEPES buffer, with pH levels ranging from 6.0 to 9.0. (C) *S*. Enteritidis was treated with the fusion ENDO‐1252/KL9P and incubated at 37°C for various time points in deionised (DI) water, with pH levels ranging from 6.0 to 9.0. (D) Time kinetics of *S*. Enteritidis treated with the fusion ENDO‐1252/KL9P in 20 mM HEPES buffer, pH levels 7.4), showed intensive growth inhibition after 3 h. More details of the assay are shown in (Supplementary Figure S1). All experiments were performed in triplicate. ND, not detected. Data represent mean ± standard deviation, and the horizontal dotted line represents the detection limit. ***p < 0.001.

*S*. Enteritidis was treated with the fusion protein ENDO‐1252/KL9P and incubated at 37°C for various time points (1, 3, 6, 9, and 12 h) under different buffer conditions (20 mM Tris–HCl, 20 mM HEPES, and autoclaved distilled water) with pH levels ranging from 6.0 to 9.0. The results indicate that ENDO‐1252/KL9P effectively inhibited the growth of *S*. Enteritidis. However, the intensity of lytic activity varied depending on the treatment duration and pH conditions (Figure 3). In 20 mM Tris–HCl, ENDO‐1252/KL9P effectively inhibited the growth of *S*. Enteritidis after 3 h at pH levels ranging from 8.0 to 9.0 (Figure 3A). In 20 mM HEPES, ENDO‐1252/KL9P exhibited intensive lytic activity after 1 h of treatment at pH 9.0 and 3 h at pH 8.0, respectively. The significant growth inhibition was also observed after 6 h of treatment at pH levels of both 7.0 and 7.4 (Figure 3B). Moreover, in autoclave distilled water conditions, intensive lytic activity was only observed after 9 h of treatment at pH levels of 8.0–9.0 (Figure 3C). Considering future applications possibility of this endolysin in livestock and humans, we selected 20 mM HEPES (pH 7.4) as the primary treatment condition to enhance the activity of the fusion protein ENDO‐1252/KL9P against *S*. Enteritidis. In addition, the results demonstrated that the ENDO‐1252/KL9P showed significant lytic activity under 20 mM HEPES (pH 7.4) after 3, 6, 9, and 12 h of incubation at 37°C (Figure 3D). Further details for the lytic activity of ENDO‐1252/KL9P under different buffer conditions are shown in Figures S1–, S3. These results support the idea that the fusion protein ENDO‐1252/KL9P could provide antimicrobial activity against *S*. Enteritidis.

### 3.3 Appropriate Dose and Temperature for the Lytic Activity of ENDO‐1252/KL9P

**FIGURE 4:**
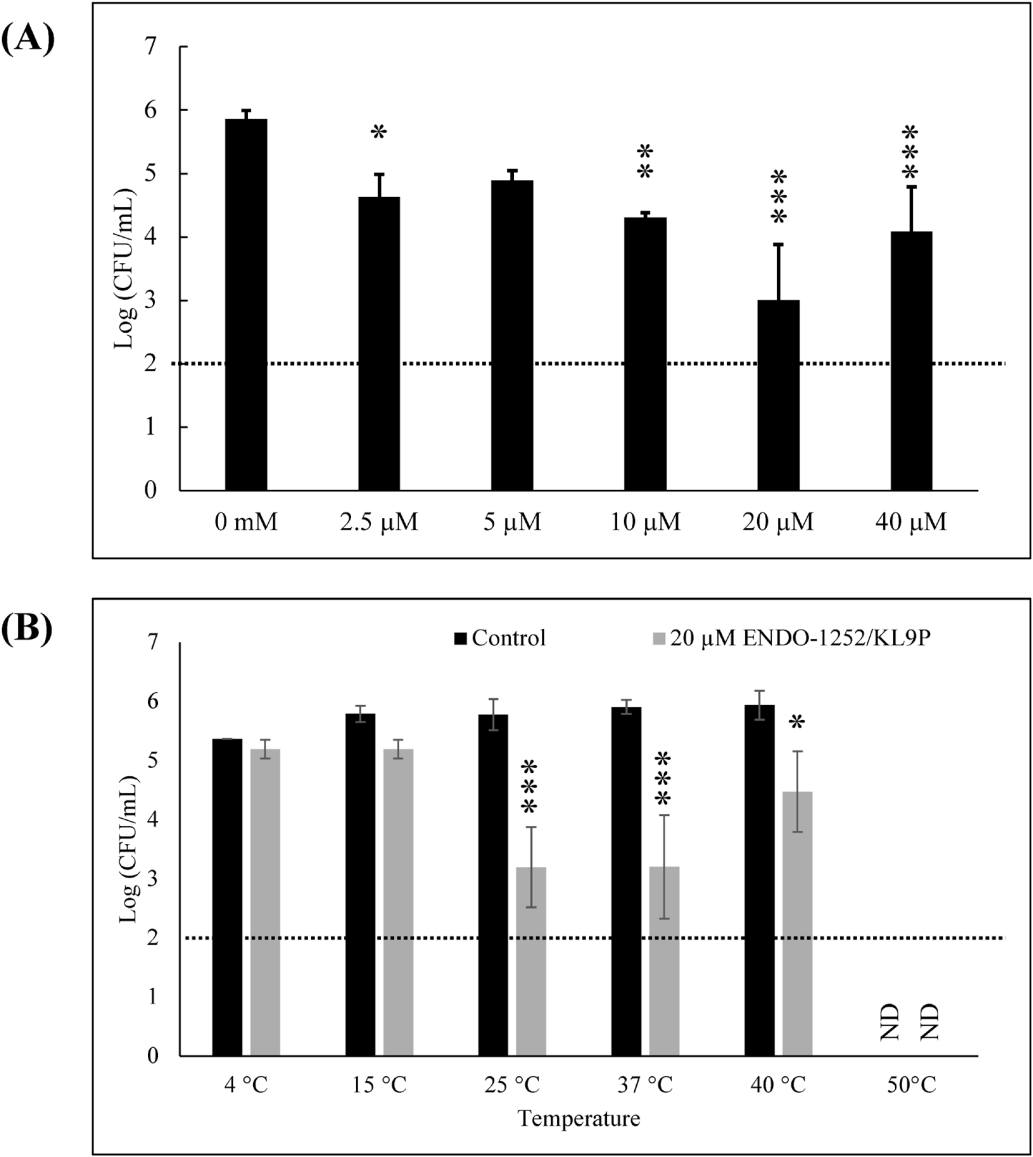
Dose‐dependent and thermal lytic activity of ENDO‐1252/KL9P against *S*. Enteritidis. (A) Dose‐dependent lytic activity of ENDO‐1252/KL9P was evaluated at concentrations of 0, 2.5, 5, 10, 20, and 40 μM in *S*. Enteritidis cultures incubated at 37°C for 6 h in HEPES buffer (pH 7.4). 20 μM ENDO‐1252/KL9P showing the most intensive lytic activity compared to untreated control group (0 uM; *p* < 0.05). Statistical significance was determined using one‐way ANOVA followed by Tukey's multiple‐comparison test. (B) Thermal lytic activity of ENDO‐1252/KL9P was examined by incubating *S*. Enteritidis cultures at 4°C, 15°C, 25°C (room temperature), 37°C, 40°C, and 50°C for 6 h, following treatment with 20 μM ENDO‐1252/KL9P in HEPES buffer (pH 7.4). Significant bacterial growth inhibition was observed at 25°C, 37°C, and 40°C (grey bars), compared to the control group treated with an elution buffer (dark black bars). At 50°C, no surviving bacteria were observed in either the treatment or control groups. Statistical significance was determined by two‐way ANOVA followed by Turkey's multiple‐comparison test for comparisons with the control group. All experiments were performed in triplicate. ND, not detected. Data represent mean ± standard deviation, and the horizontal dotted line represents the detection limit. *p < 0.05, **p < 0.01, ***p < 0.001.

Various concentrations and temperatures were evaluated to determine the optimal dose and appropriate temperature for the lytic effects of the fusion protein ENDO‐1252/KL9P. The dose‐dependent lytic activity of ENDO‐1252/KL9P was evaluated at concentrations ranging from 0, 2.5, 5, 10, 20 and 40 μM against *S*. Enteritidis at 37°C for 6 h in the presence of HEPES buffer (pH 7.4). It showed a significant growth inhibition across all treatment groups compared to the untreated control. Notably, 20 μM ENDO‐1252/KL9P exhibited the highest lytic activity against *S*. Enteritidis (Figure 4A). Therefore, 20 μM ENDO‐1252/KL9P was selected as the standard concentration for further experiments. Furthermore, the thermal stability of ENDO‐1252/KL9P was evaluated by treating *S*. Enteritidis with 20 μM ENDO‐1252/KL9P and incubating it at 4°C, 15°C, 25°C (room temperature), 37°C, 40°C and 50°C for 6 h. These findings indicated a significant growth inhibition of *S*. Enteritidis at 25°C, 37°C and 40°C, compared to the control group treated with an elution buffer (Figure 4B). Notably, at 50°C, no surviving bacteria were observed in either treatment or control groups.

### 3.4 Lytic Effects of ENDO‐1252/KL9P Against Various SE Serovars

**FIGURE 5:**
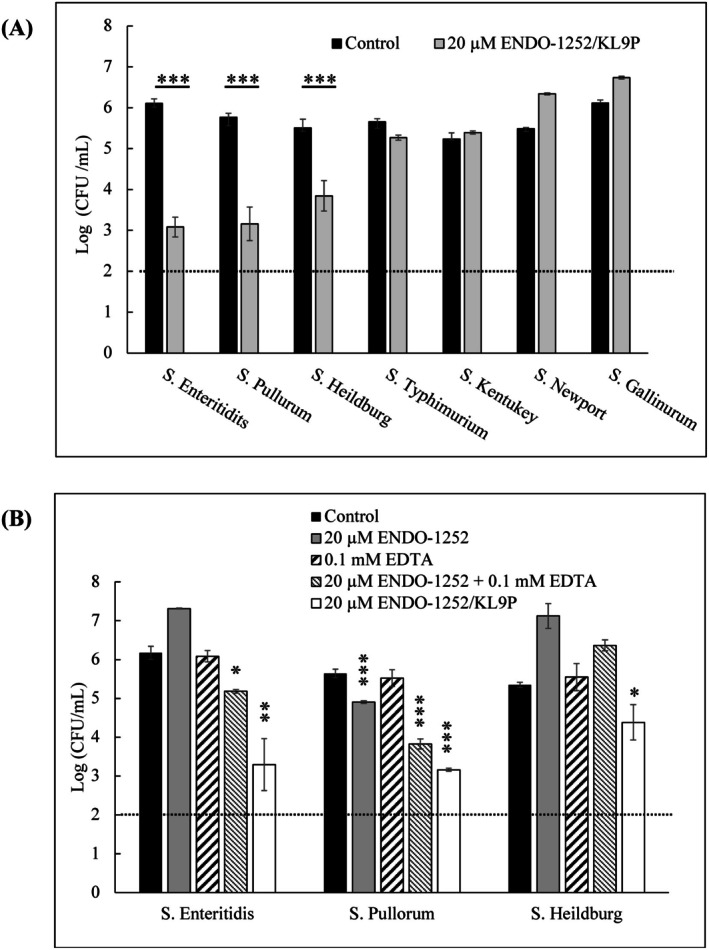
Antibacterial range of ENDO‐1252/KL9P against various SE serovars. (A) Endolysin ENDO‐1252/KL9P at a concentration of 20 μM was applied to SE serovars and incubated at 37°C for 6 h. The figure indicates that ENDO‐1252/KL9P exhibits intense lytic activity against *S*. Enteritidis, *S*. Heidelberg, and *S*. Pullorum. The blue bars represent the control group, which did not receive endolysin treatment. The dark purple bars display the results for the various *Salmonella* serovars tested. (B) Three major SE serovars (*S*. Enteritidis, *S*. Heidelberg, and *S*. Pullorum) were treated with ENDO‐1252, EDTA, a combination of ENDO‐1252 and EDTA, and the fusion protein ENDO‐1252/KL9P. The dark black bars represent the untreated control group, the dark grey bars represent serovars treated with 0.1 mM EDTA, striped bars show the results of the combination treatment, and the white bars represent treatment with the fusion protein ENDO‐1252/KL9P. All experiments were performed in triplicate. ND, not detected. Data represent mean ± standard deviation, and the horizontal dotted line represents the detection limit. All statistical significance was determined by two‐way ANOVA followed by Turkey's multiple‐comparison test for comparisons with the control group. *p < 0.05, **p < 0.01, ***P < 0.001.

To evaluate the antimicrobial effects of the fusion protein ENDO‐1252/KL9P against multiple SE serovars, we selected seven common SE serovars, including Enteritidis, Heidelberg, Pullorum, Typhimurium STLT2, Kentucky, Newport, and Gallinarum, and they were treated with 20 μM ENDO‐1252/KL9P for 6 h at 37°C. The results demonstrate the significant lytic activity of ENDO‐1252/KL9P against three serovars, including *S*. Enteritidis, *S*. Heidelberg, and *S*. Pullorum (Figure 5A). To further investigate, the lytic activity of ENDO‐1252/KL9P at different treatment conditions, specifically a synergistic effect of 20 μM ENDO‐1252 or 20 μM ENDO‐1252/KL9P in combination with 0.1 mM EDTA against the same three SE serovars (*S*. Enteritidis, *S*. Heidelberg, and *S*. Pullorum) were compared. Notably, treatment with 0.1 mM EDTA alone had minimal to no effect on bacterial growth. However, the combination of 20 μM ENDO‐1252 with 0.1 mM EDTA significantly enhanced the lytic activity of both endolysins against all tested serovars. Whereas the ENDO‐1252/KL9P fusion protein displayed the highest lytic activity of all three SE serovars, surpassing the effects of ENDO‐1252 alone or combined with EDTA (Figure 5B). These findings indicate that ENDO‐1252/KL9P is a potent antibacterial agent against multiple SE serovars, and its lytic activity is an effective measure even without synergistic outer membrane permeabilizers (OMPs).

### 3.5 Comparative Interaction of Treated *S.* Enteritidis With ENDO‐1252, ENDO‐1252/EDTA or ENDO‐1252/KL9P

**FIGURE 6:**
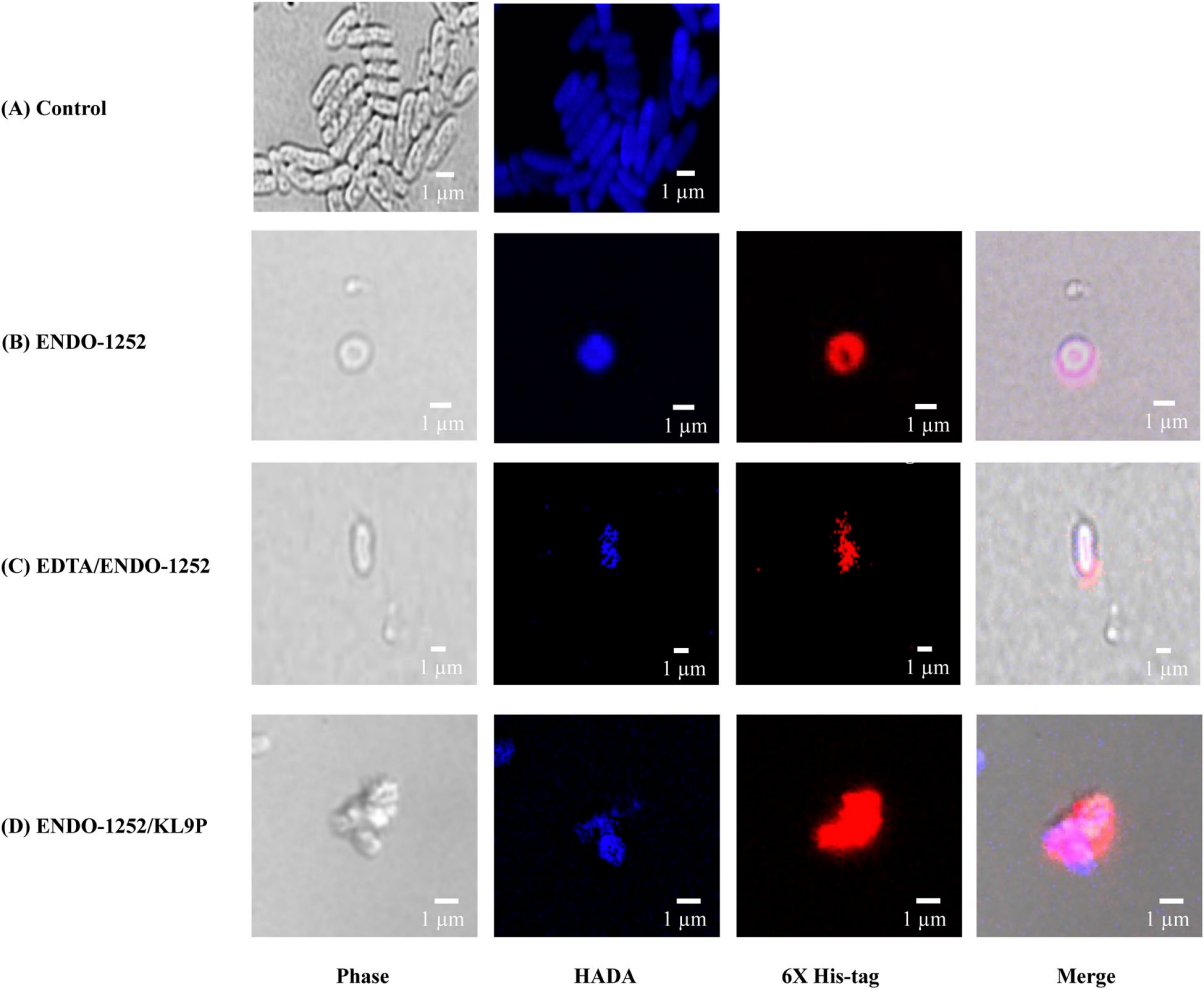
Immunofluorescence analysis of *S*. Enteritidis with ENDO‐1252, ENDO‐1252 combined with EDTA, and ENDO‐1252/KL9P. (A) HADA labels (Blue) the peptidoglycan of *S*. Enteritidis without treatment. (B) *S*. Enteritidis was treated with ENDO‐1252. (C) *S*. Enteritidis was treated with 20 μM ENDO‐1252 combined with 0.1 mM EDTA. (D) *S*. Enteritidis was treated with 20 μM ENDO‐1252/KL9P. The blue panel represents a bacterial peptidoglycan labelled by HADA. The red panel shows the 6×His‐tagged proteins (ENDO‐1252 and ENDO‐1252/KL9P). The merge panel displays the composite image of HADA and 6×His‐tagged ENDO‐1252 or ENDO‐1252/KL9P. Scale bars = 1 μm.

To evaluate the interaction of the ENDO‐1252/KL9P fusion protein with *S*. Enteritidis, bacterial cells were treated with the protein and subsequently analysed using immunofluorescence detection with an anti‐6×‐His tag antibody. HADA staining was employed to visualise the bacterial cell wall (peptidoglycan, blue), while the ENDO‐1252/KL9P fusion protein with a 6×‐His tag was detected using a fluorescently labelled secondary antibody (red). In Figure 6A, the phase contrast images (left panels) illustrate the morphology of the *S*. Enteritidis cells in each treatment. HADA staining confirmed the presence of bacterial cell walls (blue). The merged images (blue and red) show the co‐localization of the HADA‐stained cell walls and the 6×‐His tag signal. Figure 6A depicts the morphology and cell wall staining of *S*. Enteritidis without treatment. In contrast, the treatment groups, native endolysin ENDO‐1252 (Figure 6B), ENDO‐1252 combined with 0.1 mM EDTA (Figure 6C), and the ENDO‐1252/KL9P fusion protein alone (Figure 6D), demonstrate the successful binding of both the native and fusion endolysin proteins to the bacterial cell wall. These results provide further evidence in binding of this fused endolysin into the cell wall of *S*. Enteritidis and suggest that the ENDO‐1252/KL9P fusion protein effectively inhibits the growth of *S*. Enteritidis through interacting with the outer membrane of this Gram‐negative enteric pathogen.

## 4 Discussion

The increasing prevalence of antibiotic‐resistant nontyphoidal SE poses significant challenges to public health and food industries. In recent decades, the overuse and/or inappropriate use of antibiotics in both humans and animals have led to the emergence of AMR bacteria, specifically enteric bacterial pathogens, including SE (Velasquez et al. 2018). This issue has become a significant concern worldwide (Murray et al. 2022). SE can be classified as either typhoidal SE or nontyphoidal SE (Johnson et al. 2018). In the US, the major serovar of nontyphoidal SE, including *S*. Enteritidis and *S*. Typhimurium, are commonly colonised in chickens and other farm animals, which play a vital role in human salmonellosis. Centers for Disease Control and Prevention (CDC) reports nontyphoidal SE is a leading cause of foodborne outbreaks in the US (Walter et al. 2025). The transmission of nontyphoidal SE involves various routes, such as contaminated foods and water, as well as direct contact (Liu et al. 2023). Poultry and poultry products are the most critical carriers for human salmonellosis (Schoeni et al. 1995; Julianingsih et al. 2025). SE can also be transmitted between chickens and other farm animals by feed contamination or in a free‐range environment (Liu et al. 2023). Recent studies revealed that the isolates of SE were ampicillin, amoxicillin, cephradine, and many other antibiotic resistance (Velasquez et al. 2018; Gondil et al. 2020; Murray et al. 2022; Alvarado‐Martinez, Julianingsih, et al. 2023). To address this issue, many countries, including the US, have prohibited the use of sub‐therapeutic antibiotics as feed additives in response to consumer demand and to mitigate antibiotic resistance (Centner 2016; Simjee and Ippolito 2022). Therefore, more conventional poultry production has focused on alternative strategies, such as citrus oil (Julianingsih et al. 2023), plant‐derived phenolics (Alvarado‐Martinez, Tabashsum, et al. 2023), bacteriophage (Tung et al. 2023), and bacteriophage‐encoded proteins (Schmelcher et al. 2012), to prevent bacterial infection or colonisation, particularly zoonotic pathogens.

Bacteriophage‐derived muralytic proteins, known as endolysins, degrade the peptidoglycan layer of bacterial cell walls (Gondil et al. 2020; Tung et al. 2025), offering a promising alternative to traditional antibiotics (Rahman et al. 2021; Khan et al. 2024). However, their effectiveness against Gram‐negative bacteria is limited by the presence of an outer membrane, which serves as a barrier preventing endolysins from reaching the peptidoglycan layer (Loessner 2005). Some endolysins have shown intrinsic antibacterial activity against Gram‐negative bacteria without the use of outer membrane permeabilizers. LysAm24, LysECD7 and LysSi3 were effective against *Acinetobacter baumannii* (Antonova et al. 2020), while SPN9CC exhibited lytic activity against *E. coli* (Lim et al. 2014). LysPA26 (Guo et al. 2017) and LysSS (Kim et al. 2020) showed strong activity against *Pseudomonas aeruginosa* and other Gram‐negative pathogens. However, the mechanisms by which these endolysins permeate the outer membrane remain unclear (Gontijo et al. 2021). To address this barrier, a range of outer membrane permeabilizers (OMPs), such as EDTA, Triton X‐100, trichloromethane, organic acids, and the edible ε‐poly‐L‐lysine (EPL), have been utilised (Vaara 1992; Han et al. 2019; Rahman et al. 2021). These agents applied either as pretreatments or in combination with exogenous endolysins, have successfully eliminated or inhibited Gram‐negative bacteria (Gondil et al. 2020). In this study, we explored the potential of the fusion endolysin ENDO‐1252/KL9P as an alternative antimicrobial to control multiple serovars of SE. We demonstrated that the engineered endolysin ENDO‐1252/KL9P effectively interacts with outer membrane of several SE serovars and lysed various of them, which highlighted its potential role in controlling the growth and survival of SE.

In recent decades, several protein engineering strategies have been developed to address the challenge of the Gram‐negative bacterial barrier (Tung et al. 2025). However, issues such as low solubility, limited host ranges, and reduced efficiency in vivo models persist and need to be resolved (Son et al. 2023). In our previous studies, we isolated a novel bacterial strain, Salmonella bacteriophage‐1252, which can effectively inhibit the growth of multiple serovars of SE. Subsequently, we overexpressed and purified its derived endolysin, ENDO‐1252, which also exhibited strong lytic activity against several serovars of SE, including *S*. Enteritidis and *S*. Typhimurium in the presence of 0.1 mM EDTA (Tung et al. 2024).

KL‐L9P is a pro‐hinged, α‐helical amphipathic sensitizer peptide that has been shown to the relocation of anionic LPS molecules in the outer membrane of Gram‐negative bacteria, especially in *E. coli* (Hyun et al. 2020). This rearrangement allows hydrophobic antibiotics, such as rifampicin, linezolid, and clarithromycin, to pass through the membrane without disrupting or penetrating either bacterial or eukaryotic membranes, and without causing hemolytic activity (Hyun et al. 2020). A recent study also demonstrated that fusing endolysin with this sensitizer peptide Kl‐L9P, named Lys1S‐L9P, led to increased lytic activity and broader host range against Gram‐negative strains without outer membrane permeabilizers (OMPs) (Son et al. 2023). Additionally, the fusion protein showed improved stability, low cytotoxicity, and enhanced antibiofilm activity (Son et al. 2023).

In this study, fusion endolysin protein ENDO‐1252/KL9P was successfully constructed, overexpressed, and purified (Figures 1 and 2). The fusion protein ENDO‐1252/KL9P exhibited strong lytic activity in 20 mM HEPES (4‐(2‐hydroxyethyl)‐1‐piperazineethanesulfonic acid) buffer, demonstrating effective lysis effect across a range of pH from 6.0 to 9.0 (Figure 3). In this study, we also assessed other common buffers at neutral pH 7.4, including phosphate‐buffered saline (PBS), M9 buffer, LB broth, and normal saline; however, none of these buffers inhibited the growth of *Salmonella* (data not shown). These findings suggest that buffers containing salts may interfere with the lytic activity or stability of the protein. Given that 20 mM HEPES exhibited significant lytic activity at neutral pH 7.4 and is known for its excellent stability and minimal pH fluctuations in cell culture, we chose to use HEPES (pH 7.4) as the standard buffer throughout the study.

The His‐tag is primarily employed to facilitate protein purification and generally does not affect enzymatic activity. However, in some cases, His‐tags can influence protein folding, stability, or activity depending on their location and interactions with the protein structure. In this study, the His‐tag was fused at the N‐terminus, a position commonly used to minimise interference. Nevertheless, we cannot exclude the possibility that His‐tag residues may have contributed to the observed activity, as affinity tags can also interact differently depending on buffer selection (Majorek et al. 2014). Future studies need to focus on removing the His‐tag after protein overexpression and evaluating the activity and buffer interactions of the tag‐free endolysin.

Proteins are complex macromolecules with considerable variability in their folded structures. Their proper functioning, especially in aqueous environments, depends heavily on the surrounding pH. Deviations from the optimal pH range can significantly alter their functional properties (Ugwu and Apte 2004; Gupta et al. 2015). Buffers can either stabilise or destabilise proteins through their interactions. When a buffer preferentially binds to the native state of a protein, it enhances stability. Conversely, if it interacts more strongly with the denatured state, stability decreases (Ugwu and Apte 2004). Buffers may also influence protein structure by modifying the microenvironment or the water shell surrounding the protein (Ugwu and Apte 2004). As buffers play a crucial role in vitro experiment, understanding their interactions with proteins is essential. This knowledge provides insights into buffer‐induced binding effects and their influence on protein folding, refolding, and stability. In this study, we also found that higher alkaline conditions led to increased lytic activity of ENDO‐1252/KL9P across different buffer systems. This may be because an alkaline environment increases the negative charge on the bacterial out‐membrane, thereby enhancing electrostatic interactions with the positively charged regions of the amphipathic peptide (Burel et al. 2021). Consequently, this interaction may compromise membrane integrity, making the bacteria more susceptible to ENDO‐1252/KL9P (Burel et al. 2021). Further investigation is necessary to clarify the mechanisms by which HEPES enhances lytic activity at neutral and higher alkaline conditions.

Our optimization experiments identified 20 μM ENDO‐1252/KL9P in HEPES buffer (pH 7.4) as the optimal treatment condition (Figure 4A), which is critical for potential future applications in vitro and in vivo research. Although 2.5 μM ENDO‐1252/KL9P exhibited some lytic activity, 20 μM consistently demonstrated the strongest antibacterial effect. However, at even higher concentrations, we observed slightly enhance the growth of SE rather than inhibit it. This unexpected effect may be due to the tendency of protein to aggregate at high concentrations. Protein aggregation can reduce the lytic endolysin activity by masking its active sites, thereby preventing efficient degradation of the bacterial cell wall, and ultimately diminishing its antimicrobial effectiveness (Wang 2005). The broad temperature ranges from 25°C to 37°C ENDO‐1252/KL9P (Figure 4B) further indicates its suitability for various environmental conditions, making it a versatile candidate for combating *Salmonella* infections.

Interestingly, the ENDO‐1252/KL9P outperformed the native endolysin and combinations with EDTA against multiple serovars of SE, specifically *S*. Enteritidis, *S*. Heidelberg, and *S*. Pullorum (Figure 5B), demonstrating that the design of endolysin constructs can significantly impact their antimicrobial efficacy. This finding suggests that rational engineering of endolysins may provide a pathway for developing effective treatments against multiple serovars of SE.

In our previous study, the original bacteriophage ENDO‐1252 exhibited broader lytic activity and was able to lyse a wider range of *Salmonella enterica* serovars, including *S*. Typhimurium, *S*. Enteritidis, *S*. Newport, *S*. Heidelberg, *S*. Kentucky, and *S*. Gallinarum (Tung et al. 2023). This broad host range was one of the main reasons we selected this phage for further development in our study series. However, when tested independently, the phage‐derived endolysin ENDO‐1252 showed lytic activity only against *S*. Typhimurium and *S*. Enteritidis in the presence of EDTA (Tung et al. 2024). This limited activity motivated us to construct a fusion protein, ENDO‐1252/KL9P, aimed at enhancing the ability of endolysin to penetrate the outer membrane and broaden its antibacterial efficacy.

Interestingly, ENDO‐1252/KL9P exhibited activity against *S*. Enteritidis, *S*. Pullorum, and *S*. Heidelberg, but not against the other tested serovars. Differences in the cell wall of *Salmonella* serotypes are primarily attributed to variations in the O‐antigen of the LPS, which influence virulence, colony morphology, as well as host and bacteriophage specificity/interaction (Liu et al. 2014). Therefore, the observed variation in susceptibility may be due to differences in outer membrane structures among *Salmonella* strains, as well as the influence of buffer conditions on the functionality of the exogenous endolysin. The mechanisms of the exogenous endolysin interact with the outer membrane of different *Salmonella* strains remain unclear. Further investigation is required to elucidate these strain‐specific responses.

Moreover, when the SE strains were treated with ENDO‐1252 alone in HEPES buffer (pH 7.4), a slight increase in bacterial growth was observed after 6 h, rather than an inhibitory effect. In contrast, our previous study demonstrated that ENDO‐1252 exhibited mild inhibitory activity against *Salmonella Enteritidis* in Tris–HCl buffer (pH 7.4) under similar conditions (Tung et al. 2024). These findings suggest that buffer composition can significantly influence the structural conformation and functional activity of both ENDO‐1252 and ENDO‐1252/KL9P, highlighting the critical importance of buffer selection in optimising endolysin performance against Gram‐negative bacteria.

The immunofluorescence analysis confirmed the co‐localization of ENDO‐1252/KL9P with the cell wall of SE (Figure 6), reinforcing the notion that the fusion protein retains its binding capacity. This direct interaction is essential for its antimicrobial action, as effective binding facilitates the enzymatic degradation of the peptidoglycan layer. They aid in repositioning anionic LPS molecules, preventing them from blocking the penetration of antimicrobial agents. Building on this concept, we selected the peptide KL‐L9P to design a novel peptide‐fused endolysin, which extends antibacterial activity against this Gram‐negative pathogen. In Figure 6, we observed that the control group showed a higher bacterial density; however, in the other treatments, only a few cells were observed in the field of view. This result also provides visual evidence supporting the antibacterial activity of ENDO‐1252, EDTA/ENDO‐1252, and ENDO‐1252/KL9P. It is also important to consider the impact of the confocal immunofluorescence staining protocol during the study. This sample preparation procedure involves multiple washing, blocking, staining, and incubation steps, which can contribute to remove lysed or fragile cells due to compromised bacterial membranes caused by endolysin interaction. Additionally, the observed morphological changes from the rod shape to rounded or irregular shapes may be the result of cell wall damage or membrane destabilisation caused by endolysin, leading to cell collapse or structural deformation. The fusion protein ENDO‐1252/KL9P demonstrated potent antibacterial activity against multiple *Salmonella* serovars, including *S*. Enteritidis, *S*. Heidelberg, and *S*. Pullorum. By combining the bacteriophage‐encoded endolysin ENDO‐1252 with the antimicrobial peptide KL‐L9P, we successfully enhanced its lytic efficiency, overcoming the challenge posed by the outer membrane of Gram‐negative bacteria. ENDO‐1252/KL9P exhibited optimal activity at 20 mM in HEPES buffer (pH 7.4) and across a wide range of temperatures and pH levels. Additionally, the fusion protein showed superior lytic activity compared to ENDO‐1252 in combination with EDTA, without the need for outer membrane permeabilizers. These findings highlight the strong potential of using sensitizer peptides in endolysin engineering and demonstrate the promising efficacy of ENDO‐1252/KL‐L9P as a therapeutic agent against multiple serovars of SE. ENDO‐1252/KL9P is an effective alternative to conventional antibiotics for treating *Salmonella* infections and offers a promising approach to combat antibiotic resistance.

## Author Contributions

**Chuan‐Wei Tung:** writing – original draft, methodology, investigation, visualization, software, formal analysis, validation, funding acquisition, conceptualization. **Kanchan Thapa:** writing – review and editing, formal analysis. **Anna Phan:** writing – review and editing, formal analysis. **Aditi Mohapatra:** formal analysis, writing – review, and editing. **Muhammad Hashmi:** formal analysis, writing – review, and editing. **Kayla Bleich:** formal analysis, writing – review, and editing. **Debabrata Biswas:** writing – review and editing, supervision, conceptualization.

## Ethics Statement

The authors have nothing to report.

## Conflicts of Interest

The authors declare no conflicts of interest.

## Supporting information


**Figure S1:** Antimicrobial activity of the 20 μM fusion protein ENDO‐1252/KL9P at various pH levels in 20 mM Tris–HCl buffer over different time periods. (A) In 20 mM Tris–HCl (pH 6.0), ENDO‐1252/KL9P exhibited no lytic activity against *S*. Enteritidis across all time points. (B) In 20 mM Tris–HCl (pH 7.0), ENDO‐1252/KL9P exhibited no lytic activity against *S*. Enteritidis across all time points. (C) In 20 mM Tris–HCl (pH 7.4), ENDO‐1252/KL9P exhibited no lytic activity against *S*. Enteritidis across all time points. (D) In 20 mM Tris–HCl (pH 8.0), ENDO‐1252/KL9P displayed significant lytic activity against *S*. Enteritidis after 1 h, with complete eradication observed after 6 h of treatment. (E) In 20 mM Tris–HCl (pH 9.0), ENDO‐1252/KL9P displayed significant lytic activity against *S*. Enteritidis after 1 h, with no detectable *S*. Enteritidis after 3 h of treatment. All experiments were performed in triplicate. ND, not detected. Data represent the mean ± standard deviation, and the horizontal dotted line represents the detection limit. Statistical significance was determined by two‐way ANOVA followed by Tukey's multiple‐comparison test for comparisons with the control group. *p < 0.05, **p < 0.01, ***p < 0.001.

**Figure S2:** Antimicrobial activity of the 20 μM fusion protein ENDO‐1252/KL9P at various pH levels in 20 mM HEPES buffer over different time periods. (A) In 20 mM HEPES (pH 6.0), ENDO‐1252/KL9P exhibited intense lytic activity against *S*. Enteritidis across all time points. (B) In 20 mM HEPES (pH 7.0), ENDO‐1252/KL9P exhibited intense lytic activity against *S*. Enteritidis across all time points. (C) In 20 mM HEPES (pH 7.4), ENDO‐1252/KL9P exhibited intense lytic activity against *S*. Enteritidis after 3 h. (D) In 20 mM HEPES (pH 8.0), ENDO‐1252/KL9P displayed significant lytic activity against *S*. Enteritidis after 3 h, with complete eradication observed after 6 h of treatment. (E) In 20 mM HEPES (pH 9.0), ENDO‐1252/KL9P displayed significant lytic activity against *S*. Enteritidis after 1 h, with no detectable *S*. Enteritidis after 3 h of treatment. All experiments were performed in triplicate. ND, not detected. Data represent the mean ± standard deviation, and the horizontal dotted line represents the detection limit. Statistical significance was determined by two‐way ANOVA followed by Tukey's multiple‐comparison test for comparisons with the control group. *p < 0.05, **p < 0.01, ***p < 0.001.

**Figure S3:** Antimicrobial activity of the 20 μM fusion protein ENDO‐1252/KL9P at various pH levels in distil water over different time periods. (A) In distilled water (pH 6.0), ENDO‐1252/KL9P exhibited lytic activity against *S*. Enteritidis after 9 h. (B) In distilled water (pH 7.0), ENDO‐1252/KL9P exhibited lytic activity against *S*. Enteritidis after 6 h. (C) In distilled water (pH 7.4), ENDO‐1252/KL9P exhibited lytic activity against *S*. Enteritidis after 3 h. (D) In distilled water (pH 8.0), ENDO‐1252/KL9P displayed intense lytic activity after 3 h, with complete eradication observed after 12 h of treatment. (E) In distilled water (pH 9.0), ENDO‐1252/KL9P displayed intense lytic activity against *S*. Enteritidis after 3 h, with no detectable *S*. Enteritidis after 12 h of treatment. All experiments were performed in triplicate. ND: not detected. Data represent the mean ± standard deviation, and the horizontal dotted line represents the detection limit. Statistical significance was determined by two‐way ANOVA followed by Tukey's multiple‐comparison test for comparisons with the control group.  *p* < 0.05, *p < 0.01, **p < 0.001.

## Data Availability

The authors confirm that all data supporting the findings of this study are included within this article and its Supporting Information. The raw data that support the findings of this study are not openly available due to reasons of sensitivity and are available from the corresponding author upon reasonable request.
