# Supplementary figures and images for: Antibacterial Activity of a Fused Endolysin ENDO‐1252/KL9P Against Multiple Serovars of Salmonella enterica
Source: Microb Biotechnol. 2025 Oct 8;18(10):e70237. doi: 10.1111/1751-7915.70237 (PMC12508519; doi:10.1111/1751-7915.70237)

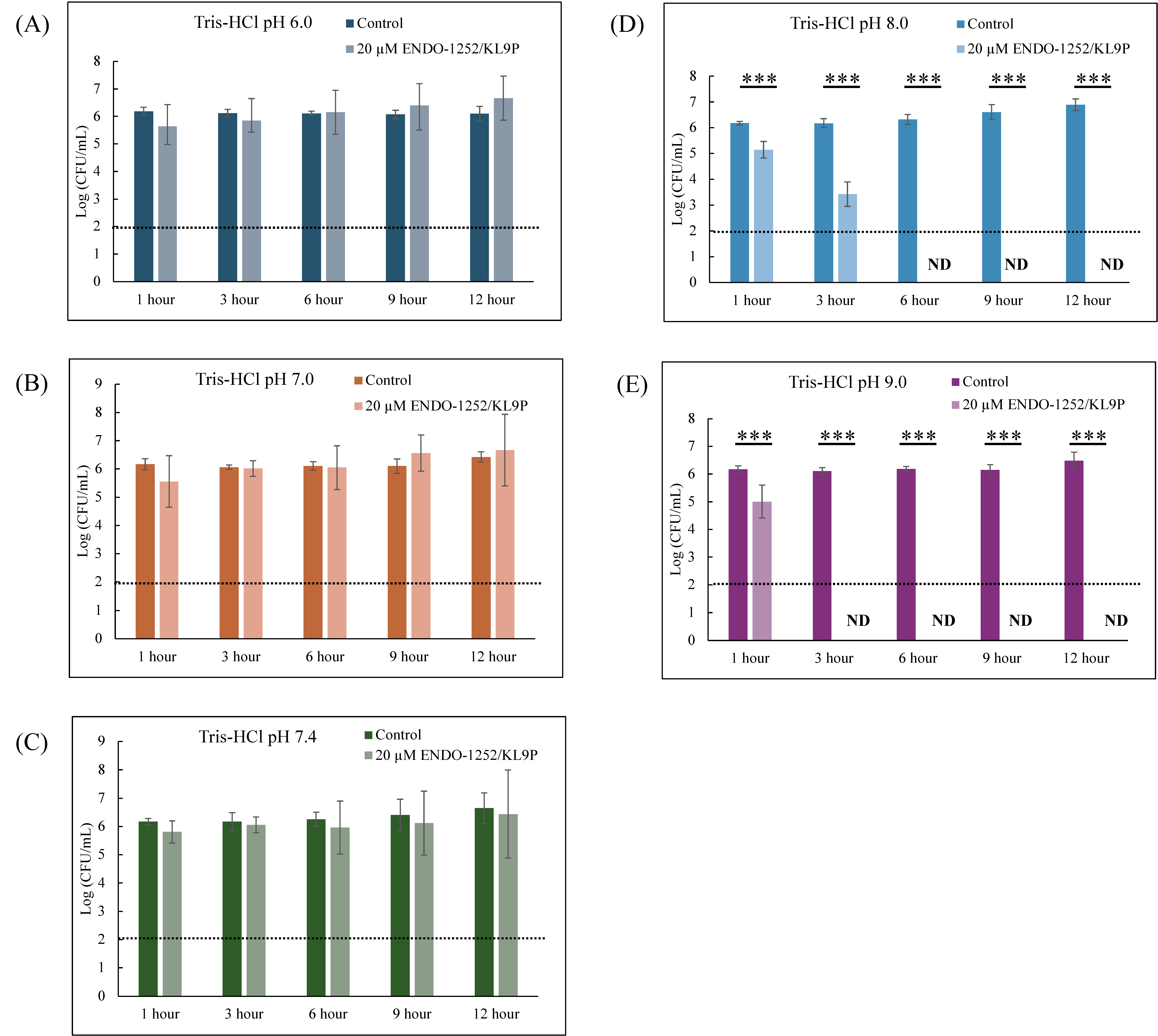

Supplement: Supplementary file 1 — Figure S1: Antimicrobial activity of the 20 μM fusion protein ENDO‐1252/KL9P at various pH levels in 20 mM Tris–HCl buffer over different time periods. (A) In 20 mM Tris–HCl (pH 6.0), ENDO‐1252/KL9P exhibited no lytic activity against S. Enteritidis across all time points. (B) In 20 mM Tris–HCl (pH 7.0), ENDO‐1252/KL9P exhibited no lytic activity against S. Enteritidis across all time points. (C) In 20 mM Tris–HCl (pH 7.4), ENDO‐1252/KL9P exhibited no lytic activity against S. Enteritidis across all time points. (D) In 20 mM Tris–HCl (pH 8.0), ENDO‐1252/KL9P displayed significant lytic activity against S. Enteritidis after 1 h, with complete eradication observed after 6 h of treatment. (E) In 20 mM Tris–HCl (pH 9.0), ENDO‐1252/KL9P displayed significant lytic activity against S. Enteritidis after 1 h, with no detectable S. Enteritidis after 3 h of treatment. All experiments were performed in triplicate. ND, not detected. Data represent the mean ± standard deviation, and the horizontal dotted line represents the detection limit. Statistical significance was determined by two‐way ANOVA followed by Tukey's multiple‐comparison test for comparisons with the control group. *p < 0.05, **p < 0.01, ***p < 0.001. [file MBT2-18-e70237-s003.tiff]

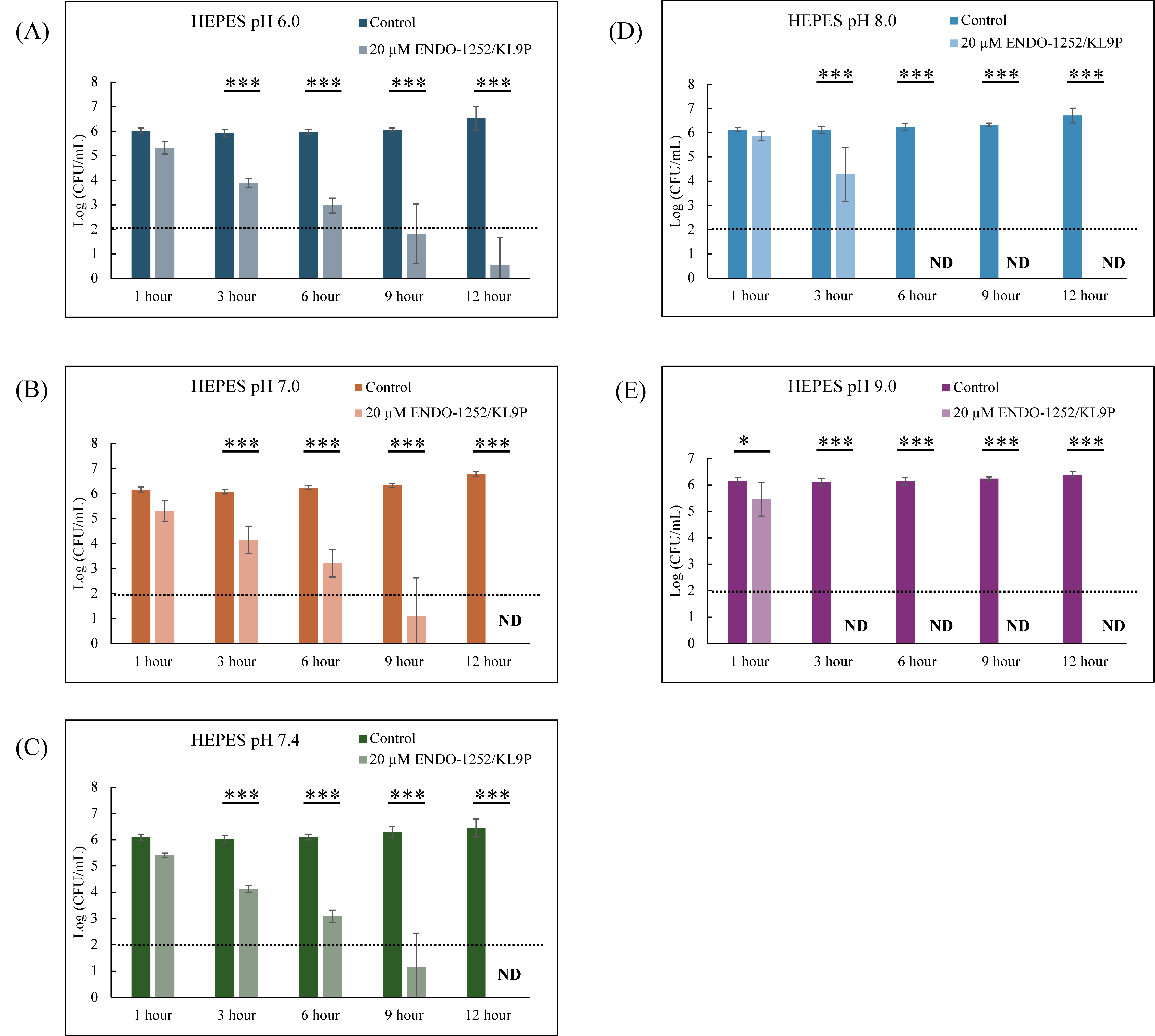

Supplement: Supplementary file 2 — Figure S2: Antimicrobial activity of the 20 μM fusion protein ENDO‐1252/KL9P at various pH levels in 20 mM HEPES buffer over different time periods. (A) In 20 mM HEPES (pH 6.0), ENDO‐1252/KL9P exhibited intense lytic activity against S. Enteritidis across all time points. (B) In 20 mM HEPES (pH 7.0), ENDO‐1252/KL9P exhibited intense lytic activity against S. Enteritidis across all time points. (C) In 20 mM HEPES (pH 7.4), ENDO‐1252/KL9P exhibited intense lytic activity against S. Enteritidis after 3 h. (D) In 20 mM HEPES (pH 8.0), ENDO‐1252/KL9P displayed significant lytic activity against S. Enteritidis after 3 h, with complete eradication observed after 6 h of treatment. (E) In 20 mM HEPES (pH 9.0), ENDO‐1252/KL9P displayed significant lytic activity against S. Enteritidis after 1 h, with no detectable S. Enteritidis after 3 h of treatment. All experiments were performed in triplicate. ND, not detected. Data represent the mean ± standard deviation, and the horizontal dotted line represents the detection limit. Statistical significance was determined by two‐way ANOVA followed by Tukey's multiple‐comparison test for comparisons with the control group. *p < 0.05, **p < 0.01, ***p < 0.001. [file MBT2-18-e70237-s001.tiff]

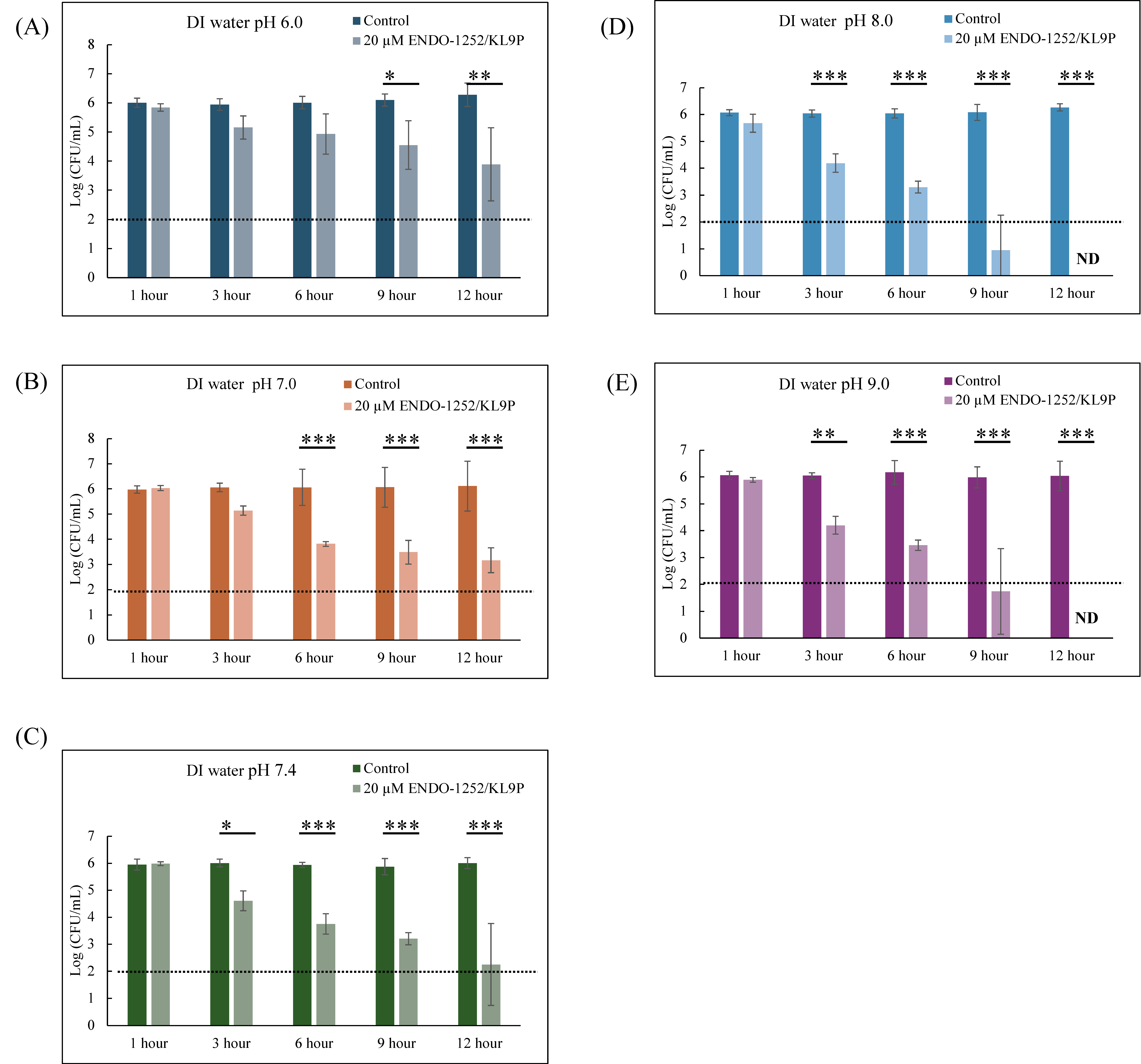

Supplement: Supplementary file 3 — Figure S3: Antimicrobial activity of the 20 μM fusion protein ENDO‐1252/KL9P at various pH levels in distil water over different time periods. (A) In distilled water (pH 6.0), ENDO‐1252/KL9P exhibited lytic activity against S. Enteritidis after 9 h. (B) In distilled water (pH 7.0), ENDO‐1252/KL9P exhibited lytic activity against S. Enteritidis after 6 h. (C) In distilled water (pH 7.4), ENDO‐1252/KL9P exhibited lytic activity against S. Enteritidis after 3 h. (D) In distilled water (pH 8.0), ENDO‐1252/KL9P displayed intense lytic activity after 3 h, with complete eradication observed after 12 h of treatment. (E) In distilled water (pH 9.0), ENDO‐1252/KL9P displayed intense lytic activity against S. Enteritidis after 3 h, with no detectable S. Enteritidis after 12 h of treatment. All experiments were performed in triplicate. ND: not detected. Data represent the mean ± standard deviation, and the horizontal dotted line represents the detection limit. Statistical significance was determined by two‐way ANOVA followed by Tukey's multiple‐comparison test for comparisons with the control group. p < 0.05, *p < 0.01, **p < 0.001. [file MBT2-18-e70237-s002.tiff]
